# Cylindrical vector beam multiplexer/demultiplexer using off-axis polarization control

**DOI:** 10.1038/s41377-021-00667-7

**Published:** 2021-11-02

**Authors:** Shuqing Chen, Zhiqiang Xie, Huapeng Ye, Xinrou Wang, Zhenghao Guo, Yanliang He, Ying Li, Xiaocong Yuan, Dianyuan Fan

**Affiliations:** 1 Institute of Microscale Optoelectronics, Shenzhen University, 518060 Shenzhen, China; 2 Guangdong Provincial Key Laboratory of Optical Information Materials and Technology & Institute of Electronic Paper Displays, South China Academy of Advanced Optoelectronics, South China Normal University, 510006 Guangzhou, China

**Keywords:** Nanophotonics and plasmonics, Fibre optics and optical communications

## Abstract

The emergence of cylindrical vector beam (CVB) multiplexing has opened new avenues for high-capacity optical communication. Although several configurations have been developed to couple/separate CVBs, the CVB multiplexer/demultiplexer remains elusive due to lack of effective off-axis polarization control technologies. Here we report a straightforward approach to realize off-axis polarization control for CVB multiplexing/demultiplexing based on a metal–dielectric–metal metasurface. We show that the left- and right-handed circularly polarized (LHCP/RHCP) components of CVBs are independently modulated via spin-to-orbit interactions by the properly designed metasurface, and then simultaneously multiplexed and demultiplexed due to the reversibility of light path and the conservation of vector mode. We also show that the proposed multiplexers/demultiplexers are broadband (from 1310 to 1625 nm) and compatible with wavelength-division-multiplexing. As a proof of concept, we successfully demonstrate a four-channel CVB multiplexing communication, combining wavelength-division-multiplexing and polarization-division-multiplexing with a transmission rate of 1.56 Tbit/s and a bit-error-rate of 10^−6^ at the receive power of −21.6 dBm. This study paves the way for CVB multiplexing/demultiplexing and may benefit high-capacity CVB communication.

## Introduction

Multiplexing, which coaxially transmits multiple signal channels, has a significant importance for increasing the optical communication density^1–4^. Driven by the wavelength-division-multiplexing, the transmission rate of optical communication has been incredibly increased to Tbit/s^5–7^. However, further enhancement of the transmission rate is facing limited availability in bandwidth if only relying on adding spectrum multiplexed channels. Beyond using more spectra, the two orthogonal polarization states have also been explored for multiplexing^8–10^. So far, the development of high-speed optical communication is hindered by the lack of suitable multiplexing dimensions. Recently, cylindrical vector beam (CVB) multiplexing has emerged as a powerful technique to boost signal channels^11–17^. The CVBs enable robust transmission ability in atmospheric turbulence due to its inherent spatially inhomogeneous polarization distribution and ability to transmit over ultra-long distance because the vector mode is the eigenmodes of few-mode fiber^18–22^. More importantly, CVB multiplexing is highly promising for optical communication compatible with conventional wavelength-division-multiplexing and polarization-division-multiplexing as it is independent of wavelength and polarization states.

Coupling and separating CVBs are two pivotal elements in CVB multiplexing communication. Although a large variety of devices have been proposed to modulate homogeneous light beams^23–27^, such as Q-plate, spatial light modulator (SLM), and spiral phase plate, the modulation of light beams with spatially inhomogeneous polarization distribution remains challenging. So far, the spin-dependent Pancharatnam–Berry (P-B) phase devices have been explored to separate CVBs^28,29^. The mechanism is to independently control the wavefront of the respective left- and right-handed circularly polarized (LHCP/RHCP) components as CVB can be theoretically decomposed into two vortex beams with opposite-handed circular polarization and conjugate topological charges. However, although this approach can linearly separate the CVBs, the coordinate transformation destroys the polarization structure of the CVB, rendering it only effective for CVB demultiplexing^30–32^. Alternatively, off-axis control technologies such as miniature Dammann vortex gratings have also been investigated to couple and separate light beams^33^. However, it is usually limited to light beams with homogeneous polarization due to its phase-only grating structure. For CVBs with inhomogeneous polarization, a gradient phase device is required to create a gradient phase difference between the LHCP and RHCP components, so that the CVBs with off-axis incident angles can coaxially propagate and carry different phase structures. Despite considerable efforts of off-axis control technologies^34–36^, the off-axis polarization control of CVBs still remains a challenge.

In this work, we report a straightforward approach to realize off-axis spin-independent polarization control for CVB multiplexing/demultiplexing based on a plasmonic metasurface. By combining the P-B phase with the propagation phase^37–42^, we show that off-axis control of polarization is realized with metal–dielectric–metal metasurface, which is consisting of subwavelength Au nanoantenna on a SiO_2_-Au-Si substrate. In principle, the linearly polarized Gaussian beams with different incident angles are first transferred into coaxially transmitted CVBs, subsequently reflected, and then spatially separated by the metasurface. Hence, the CVB with inverse polarization order is recovered to the fundamental mode for demultiplexing. We also show that the multiplexers/demultiplexers are broadband (working wavelength ranging from 1310 to 1625 nm) and compatible with wavelength-division-multiplexing. Furthermore, as a proof of concept, we successfully demonstrate a four-channel CVB multiplexing communication with four vector mode channels (*m* = ±1, ±2), combining wavelength-division-multiplexing and polarization-division-multiplexing with a transmission rate of 1.56 Tbit/s and a bit-error-rate (BER) of 10^−6^ at the received power of −21.6 dBm.

## Results

### Principle of off-axis polarization control

**Fig. 1:**
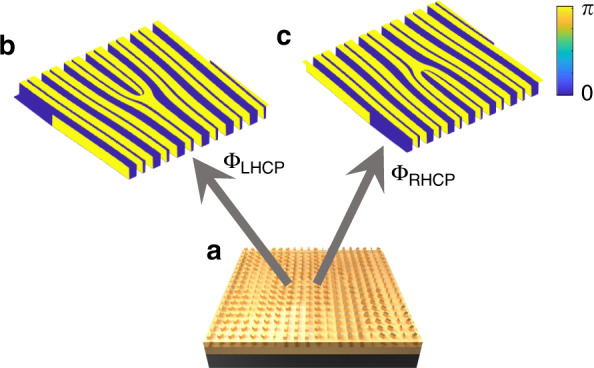
Schematic of off-axis polarization control based on spin-independent phase modulation. **a** Device for off-axis polarization control. **b**, **c** Phase response of LHCP and RHCP components

CVB, which possesses spatially inhomogeneous polarization distribution, is the axially symmetric solution to the full vector electromagnetic wave equation^43–45^. Due to the polarization singularity of CVB, it inherently has a null field in the beam center. The Jones matrix of the CVB with *m*-th polarization order can be defined by:

1 $$\begin{array}{lll}E_{{\rm{vector}}} = E_0\left[ {\begin{array}{*{20}{l}} {\cos (m\theta + \varphi _0)} \\ {\sin (m\theta + \varphi _0)} \end{array}} \right]\\\quad\quad\;\; = E_0\left[ {\begin{array}{*{20}{l}} {\frac{1}{2}\exp [i(m\theta + \varphi _0)] + \frac{1}{2}\exp [ - i(m\theta + \varphi _0)]} \\ {\frac{1}{2}\exp [i(m\theta + \varphi _0)] - \frac{1}{2}\exp [ - i(m\theta + \varphi _0)]} \end{array}} \right]\\ \quad\quad\;\;= \frac{1}{2}E_0\exp [i(m\theta + \varphi _0)]\left[ {\begin{array}{*{20}{l}} 1 \\ { - i} \end{array}} \right] + \frac{1}{2}E_0\exp [ - i(m\theta + \varphi _0)]\left[ {\begin{array}{*{20}{l}} 1 \\ i \end{array}} \right]\end{array}$$

where *E*_0_ is the simplified amplitude, *m* is the polarization order, *θ* is the azimuthal angle, and *φ*_0_ is the initial phase. From Eq. (1), it can be seen that CVB can be considered as the linear superposition of RHCP vortex beam with helical phase of exp(*imθ*) and LHCP vortex beam with helical phase of exp(−*imθ*). More specifically, the two vortex beams have opposite topological charges and orthogonal circular polarizations. For example, the CVB (*m* = +2) is the linear superposition of a RHCP vortex beam with the topological charge of +2 and a LHCP vortex beam with the topological charge of −2. To realize off-axis control of CVB, two different grating phases corresponding to the RHCP and LHCP components are demanded. As shown in Fig. 1b, c, for the LHCP component, the transmission function can be expressed as:

2 $${\Phi}_{{\rm{LHCP}}} = \mathop {\sum}\limits_{n = - \frac{N}{2}}^{n = \frac{N}{2}} {C_n\exp \left[ {in \left(\frac{{2\pi x}}{T} + {\Delta}m\theta \right)} \right]} \left[ {\begin{array}{*{20}{c}} 1 \\ { - i} \end{array}} \right]$$

where Δ*m* is the interval of topological charges, *T* is the period of grating, *n* is the diffraction order from $- N/2$ to $N/2$, and $\left| {C_n} \right|^2 = \,1/N$ is the power of the *n*-th order normalized with reference to the total power. Similarly, for the RHCP component, it can be expressed as:

3 $${\Phi}_{{\rm{RHCP}}} = \mathop {\sum}\limits_{n = - \frac{N}{2}}^{n = \frac{N}{2}} {C_n\exp \left[ {in\left(\frac{{2\pi x}}{T} - {\Delta}m\theta \right)} \right]} \left[ {\begin{array}{*{20}{c}} 1 \\ i \end{array}} \right]$$

If a linearly polarized Gaussian beam is incident, the transmission function of off-axis polarization control can be described as:

4 $${\Phi}_{{\rm{LP}}} = \mathop {\sum}\limits_{n = - \frac{N}{2}}^{n = \frac{N}{2}} {C_n\exp \left(in\frac{{2\pi x}}{T}\right)} \left[ {\begin{array}{*{20}{c}} {\cos ((n \times {\Delta}m)\theta )} \\ {\sin ((n \times {\Delta}m)\theta )} \end{array}} \right]$$

In principle, to achieve off-axis polarization control, the LHCP(RHCP) component should be first converted into RHCP(LHCP) by using spin-dependent phase device and then independently modulated without conjugacy. Here it should be pointed out that the polarization control cannot be accomplished because the phase differences of the conjugate phase of the binary grating are the same (see Supplementary Note 2).

### Realization of off-axis polarization control based on compound-phase metasurface

**Fig. 2:**
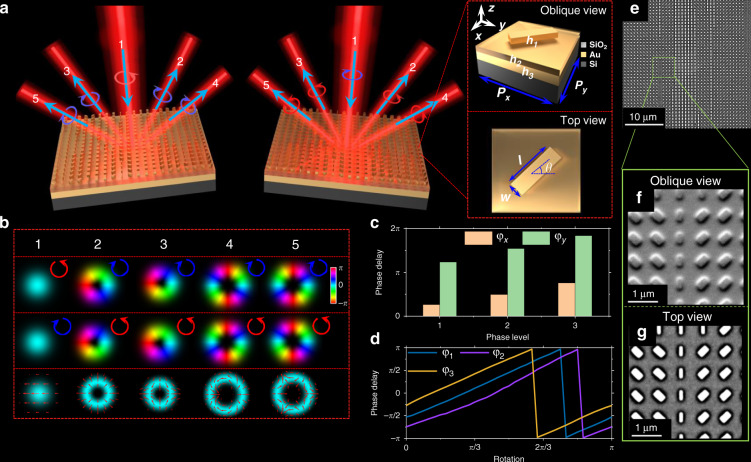
Design and realization of metasurface-based CVB multiplexer/demultiplexer. **a** Schematic illustration of generating CVBs, the blue circular arrow represents RHCP, and red is LHCP. The oblique and top views of a unit structure are shown in the red dotted frame, the unit cell dimensions thickness *h*_1_ = 50 nm, and period *P*_*x*_ = *P*_*y*_ = 800 nm that are deposited on SiO_2_ layer with thickness *h*_2_ = 150 nm and Au mirror thickness *h*_3_ = 200 nm. **b** The light field distribution at each position in **a**. **c** Phase of *x*- and *y*-direction of the metasurface, here we divide 0 to $\pi$ into 3 levels. **d** Calculated phase delay of three size nanoantenna for LHCP beam input. **e**–**g** Scanning electron microscopy (SEM) images of fabricated metasurface, **e** large-scale view, **f** oblique view, **g** top view of structures

The working principle of metasurface-based CVB multiplexer/demultiplexer is schematically illustrated in Fig. 2. To uniformly distribute light intensity in the far-field, we adopt the binarized Dammann vortex grating phase, where the position and number of phase-jump points in the normalized period are determined by the diffraction orders (see Supplementary Note 1). The grating periods (*T*) are the same between RHCP and LHCP phase response, but the topological charge intervals are different, which are shown in Fig. 2a. The transmission function of LHCP and RHCP are ${\Phi}_{{\rm{LHCP}}} = \mathop {\sum}\limits_{n = - 2}^{n = 2} {C_n\exp \left[ {in(\frac{{2\pi x}}{T} + \theta )} \right]}$ and ${\Phi}_{{\rm{RHCP}}} = \mathop {\sum}\limits_{n = - 2}^{n = 2} {C_n\exp \left[ {in(\frac{{2\pi x}}{T} - \theta )} \right]}$, respectively. The far-field intensity distributions of different polarized incident light beams are shown in Fig. 2b. If a LHCP Gaussian beam is incident on the metasurface, four RHCP vortex beams can be obtained at the diffraction orders with topological charges of *l* = −2, −1, +1, +2, respectively. If a RHCP Gaussian beam is incident, the beams at these diffraction orders will be replaced by LHCP vortex beams with *l* = +2, +1, −1, −2. Hence, if a linearly polarized Gaussian beam (contain both LHCP and RHCP components) is incident, it leads to four CVBs with orders of *m* = −2, −1, +1, +2. However, traditional P-B phase elements perform the transformation of $\left| L \right\rangle \to {\mathop{\rm e}\nolimits} ^{i2\theta }\left| R \right\rangle$ and $\left| R \right\rangle \to {\mathop{\rm e}\nolimits} ^{ - i2\theta }\left| L \right\rangle$, where *θ* is the half-wave (*π*) retardance rotated angles, and the LHCP/RHCP components are converted to carry opposite spin state and conjugate phase. Hence, P-B phase elements cannot independently modulate the LHCP and RHCP components, which means that vortex beam with an opposite topological charge at the same diffraction order cannot be achieved. To solve this problem, we propose to independently modulate the LHCP and RHCP components by combining the propagation phase and P-B phase (see Supplementary Note 3). Since the spin transformation from LHCP to RHCP is still needed, we employ the unit structure with half-wave plate effect ($\varphi _x\,-\,\varphi _y\,=\,\pi$, where $\varphi _x$ and $\varphi _y$ are the propagation phase on the *x*- and *y*-direction linear polarization, respectively). To achieve $\left| L \right\rangle \to {\mathop{\rm e}\nolimits} ^{i\phi _1}\left| R \right\rangle$ and $\left| R \right\rangle \to {\mathop{\rm e}\nolimits} ^{i\phi _2}\left| L \right\rangle$, the phase modulation of metasurface at each point should satisfy:

5 $$\exp (i\phi _1(x,y)) = \exp (i\varphi _x(x,y))\exp (i2\psi (x,y))$$

6 $$\exp (i\phi _2(x,y)) = \exp (i\varphi _x(x,y))\exp ( - i2\psi (x,y))$$

where $\varphi _x(x,y)$ represents the propagation phase, and $\pm i2\psi (x,y)$ is the P-B phase. Therefore, we can get $\varphi _x(x,y) = (\phi _1(x,y) + \phi _2(x,y))/2$, $\psi (x,y) = (\phi _1(x,y) - \phi _2(x,y))/4$, where $\psi (x,y)$ is the orientation angle of optical axis of structural units. To experimentally realize the CVB multiplexer/demultiplexer, we use the subwavelength Au nanoantennas with a fixed height of 50 nm while varied length, width, and rotation angle. As shown in the red dotted frame of Fig. 2a, the proposed metasurface is composed of sandwiched structure. To be specific, from the top to the bottom, they are Au nanoantennas with thickness of 50 nm, silica film with 200 nm thickness, gold film with 150 nm thickness, and silicon wafer. The lattice constant is set to 800 nm, and the length and width of Au nanoantennas are *l* and *w*, respectively. The height of nanoantennas and the lattice constant are selected by comprehensively considering the fabrication accuracy of the processing equipment and the optimal performance of the metasurface after sweeping the geometrical parameters of the unit cell. The Au is chosen as the building material of the metasurface due to its high reflectivity in a broadband from visible to infrared. The demanded propagation phase of *φ*_*x*_ and *φ*_*y*_ are shown in Fig. 2c. To meet the half-wave retardance, the phase difference between *φ*_*x*_ and *φ*_*y*_ is a constant *π*. In this case, the phase delay induced by the P-B phase is given by $\exp ( \pm i2\psi (x,y))$. As shown in Fig. 2d, *φ*_1_, *φ*_2_, and *φ*_3_ are the phase delay of three metallic nanoantennas with different orientation angles for LHCP beams. This metasurface-based multiplexer/demultiplexer is fabricated on a SiO_2_-Au-Si wafer with a 150 nm-thick PMMA layer by standard electron beam lithography technology. The scanning electron microscopy (SEM) images are shown in Fig. 2e, and the oblique and top view are shown in Fig. 2f, g. The detailed fabrication process is described in the “Materials and methods” section.

### Performance of metasurface-based off-axis polarization control

**Fig. 3:**
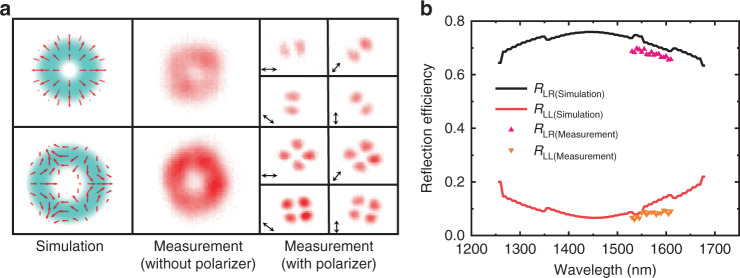
Optical characterization of the metasurface-based CVB multiplexer/demultiplexer. **a** Left: simulated optical intensity distribution of the CVB at different diffraction order (*m* = +1 and *m* = −2); middle and right: measured intensity profiles are shown both with and without a linear polarizer placed in front of the camera. **b** Simulated and measured reflection coefficients *R*_LR_ and *R*_LL_ for circularly polarized light

The characteristics of the produced CVBs have been experimentally investigated, where the experimental set-up is schematically shown in Supplementary Note 4. The far-field intensity patterns of different polarized incident beams are captured by a near-infrared camera, which are shown in Fig. 3a. When a linearly polarized Gaussian beam is incident, the CVBs with the polarization orders from −2 to +2 at corresponding diffraction orders are obtained. As shown in Fig. 3a, the left column shows the theoretical optical intensity and polarization distributions at the diffraction orders of +1 and −2, where the red arrow represents the polarization direction at the beam cross-section. The measured intensity and polarization distributions are shown in the middle and right columns of Fig. 3a. The polarization distributions are measured by a linear polarizer (LP) with different rotation angles in front of the camera. The black double-headed arrows show the direction of the polarizer’s transmission axis. After filtered by LP, the CVB is decomposed into several side lobes. The number of side lobes depends on the polarization order of CVBs (polarization order is half of the number of side lobes), which rotate with the transmission axis of the LP (the polarization order is positive with the side lobe rotating in the same direction as the optical axis, otherwise it is negative). Based on the number and the rotation direction of side lobes, the polarization orders of the CVBs are *m* = +1, −2, which is consistent with theoretical expectations. We also verified the broadband performance of the metasurface. The reflection coefficients for the circularly polarized light beam *R*_LR_ and *R*_LL_ are simulated with the finite-difference time-domain (FDTD) method, where *R*_LL_ and *R*_LR_ represent the proportions of LHCP and RHCP in the output beam (when the incident beam is LHCP beam), respectively. As shown in Fig. 3b, the metasurface’s working bandwidth is between 1260 and 1675 nm. The scatter plots of Fig. 3b are the measured reflection coefficients of *R*_LR_ and *R*_LL_ from 1529 to 1605 nm. The slight disagreement between theoretical and experimental results might arise from the experimental errors, including the metasurface fabrication and optical characterization. Furthermore, we measured the metasurface’s far-field distribution at the working wavelength of 1310 nm (see Supplementary Note 5). The results indicate that the metasurface can also perform CVB multiplexing/demultiplexing at $\lambda = 1310\, {\rm{nm}}$.

### CVB multiplexing communication

**Fig. 4:**
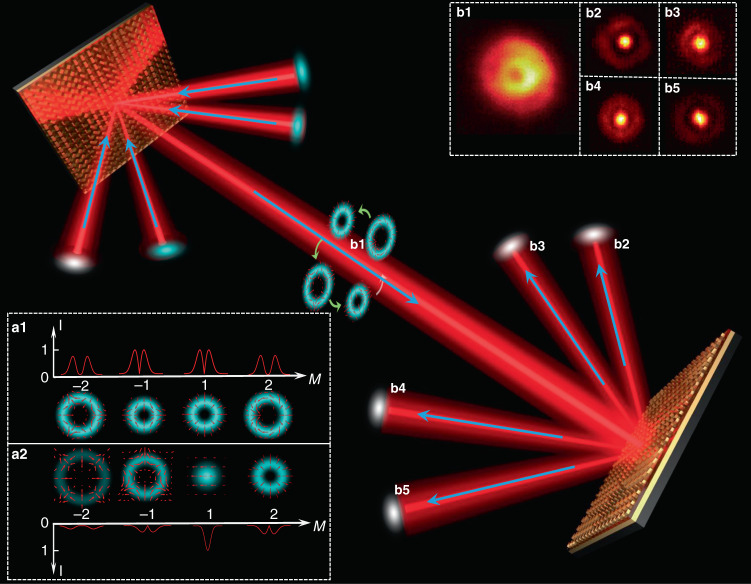
Schematic of CVB modes multiplexing/demultiplexing by using metasurface-based Dammann optical vector gratings. **a1** Polarization and intensity distributions in each diffraction order with Gaussian beam incident, respectively. **a2** Polarization and intensity distributions in each diffraction order with CVB (*m* = −1) incident. **b1** Measured coaxial CVB with 4 modes ($m = \pm 1,{{{\mathrm{ }}}} \pm 2$); **b2**−**b5** measured Gaussian points after demultiplexing of the different CVB mode channels

We employ two metasurfaces as CVB multiplexer and demultiplexer, and the multiplexing/demultiplexing experiment is schematically depicted in Fig. 4. Four Gaussian beams carrying digital signals are incident on the multiplexer at the angles of different diffraction orders (which are labeled as ±1 and ±2). All the incident Gaussian fundamental modes are transformed into CVBs with different polarization orders (dependent on incident angle) and coaxially transmitted along with the zeroth diffraction order. For signal demodulation, according to our previous works^40–42^, the CVB with polarization order (−*m*) can be recovered to the fundamental spatial mode by using the corresponding metasurface, which can also be used to generate *m*-th CVB. Figure 4a1, a2 shows the schematic illustration of the conversion from CVB to fundamental spatial mode. If a Gaussian beam is incident, the CVBs with the polarization orders from −2 to +2 are obtained at different diffraction orders, and *M* represents the diffraction order in Fig. 4a. The polarization and intensity distributions of the CVBs with different orders are shown in Fig. 4a1. The beam radius is proportional to the absolute value of its polarization order. If a CVB with *m* = −1 is incident, a Gaussian beam is obtained at the diffraction order of +1 (Fig. 4a2). In addition, the polarization order of CVB in other diffractive order is changed. For example, in the diffraction order of *M* = −2, the polarization order of CVB transforms from *m* = −2 to *m* = −3. Hence, at the receiver, the coaxially transmitted CVBs are demultiplexed into spatially separated Gaussian beams by the metasurface. Figure 4b1–b5 shows the measured intensity distributions of the multiplexed coaxial beam and demultiplexed CVBs at each diffraction orders. Owing to the vector mode conservation, only the vector mode with inverse polarization order is recovered to fundamental spatial mode with a relatively stronger intensity distribution at the center, which is filtered out by a fundamental mode filter like an aperture. Finally, the recovered fundamental spatial modes are coupled into optical fibers for signal detection.

**Fig. 5:**
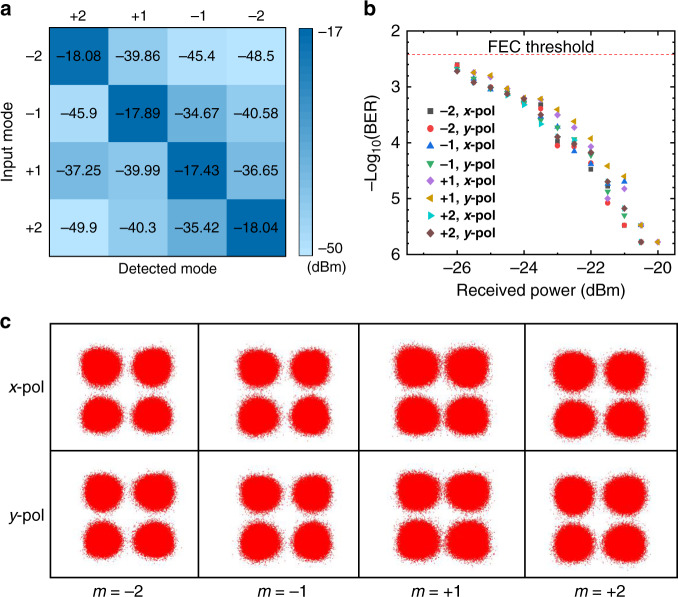
Experimental results are used to prove that the metasurface-based CVB mode multiplexer/demultiplexer is compatible with polarization multiplexing. **a** Measured mode crosstalk of four CVB mode channels. **b** BERs corresponding to eight CVB mode channels at 1554 nm. **c** Constellations of the QPSK signals of CVB modes (*m* = ±1, ±2) with *x*-pol and *y*-pol, respectively

As a proof of concept, we utilize these metasurfaces to multiplex and demultiplex the CVBs with *m* = ±1, ±2 (see Supplementary Note 6). For the incident *x*-polarized and *y*-polarized Gaussian beam, the radial and azimuthal CVB can be obtained due to the orthogonality of *x*- (*x*-pol) and *y*-direction linear polarization (*y*-pol). The radial and azimuthal CVB are also orthogonal. Hence, polarization-division-multiplexing and mode-division-multiplexing can be combined. We realized four CVB modes multiplexing and demultiplexing at the wavelength of 1554 nm by combining polarization-division-multiplexing, which carry 400 Gbit/s quadrature phase shift keying (QPSK) signals. The light intensity matrix of the four demultiplexed CVBs ($m{{{\mathrm{ = }}}} \pm {{{\mathrm{1,}}}} \pm {{{\mathrm{2}}}}$), which were applied in the communication system, is shown in Fig. 5a.

The diagonal of the matrix is the intensity of the optical signal obtained by demodulation, and the others are crosstalk. The crosstalk relates to the difference in polarization orders between adjacent channels. Here we set the interval between adjacent channels to ${\Delta}m = 1$, and the crosstalk between adjacent channels can be reduced by increasing ${\Delta}m$. Hence, the BER performance of this communication system can be further improved. Figure 5b represents the BERs of the CVB channels at 1554 nm. “−2, *x*-pol” represents the channel is *x*-polarization input and polarization order is $m = - 2$, and the others can be done similarly. The BERs are all below the hard-decision forward-error-correction (FEC) threshold of $3.8 \times 10^{ - 3}$. These results help to confirm that the metasurfaces can realize low-crosstalk and high-speed CVB mode multiplexing/demultiplexing.

We further construct a CVB mode multiplexing communication system, which combines wavelength-division-multiplexing and polarization-division-multiplexing. The optical spectra at the wavelengths of 1551.72, 1553.33, 1554.94, and 1556.55 nm are measured before and after multiplexing (see Supplementary Note 7). It can be noticed from Fig. S7 that the linewidth of resonant peaks is broadened as the power of signal is amplified by Erbium-doped fiber amplifier. However, the peak wavelength remains unchanged after propagating through the metasurface, indicating that the metasurface is nondispersive over certain bandwidth. Moreover, we test the BERs of the CVB channels with different vector modes and wavelengths to analyze data signals. The BERs of 32 channels, including 4 wavelengths, 2 polarizations, and 4 CVB modes, are measured.

**Fig. 6:**
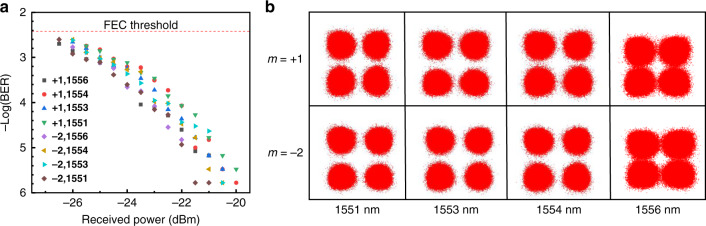
Demonstration of the CVB mode multiplexer/demultiplexer is compatible with wavelength multiplexing. **a** Measured BERs of two CVB modes at four wavelengths. **b** Constellations of QPSK signals carried by CVB modes (*m* = −2, *m* = +1) at different wavelengths (1551, 1553, 1554, and 1556 nm

As shown in Fig. 6a, we select eight channels to analyze the communication performance. “+1, 1556” represents the CVB channel at the wavelength of 1556 nm with $m = + 1$, and the rest is done in the same manner. It can be found that the BERs are all below the FEC threshold. When the received power reaches −19 dBm, there is almost no BER. Figure 6b depicts the constellations of CVB channels ($m = - 2, + 1$) with the wavelengths of 1551, 1553, 1554, and 1556 nm (at the receiver power of −22 dBm). The constellations at 1551, 1553, 1554, and 1556 nm are similar at different CVB modes, and the constellations of different CVB modes at the same wavelength are also similar, which demonstrate that the received crosstalk of each channel are equivalent, and the communication system performance is reliable. Furthermore, it should be noted that all channels were individually modulated, detected, and simultaneously analyzed in real time. These results indicate that the metasurface is effective in CVB multiplexing communication.

## Discussion

Coupling and separating CVB modes are two critical procedures in multiplexing/demultiplexing, where off-axis manipulation of CVBs is highly demanded. To control the light beams off-axially, the gradient phase or Dammann vortex grating is usually employed to manipulate the wavevector based on phase modulation^46–49^. However, these methods are no longer effective for CVBs due to its inhomogeneous polarization states. According to Jones matrix analysis, CVB can be obtained by linearly superposing two orthogonal circularly polarized vortex beams with conjugate topological charges. After decomposing into LHCP and RHCP components, CVBs can be off-axis controlled by independently modulating the phase of these two components, and thus the mode coupling and separating can be achieved via introducing gradient phase changes. Although a spin-multiplexing metasurface-based Dammann vortex grating can be utilized to measure the phase and polarization singularities of light by using detour phase^50^, the two spin components should be essentially projected into two opposite positions, which is incapable of CVB mode multiplexing/demultiplexing.

We independently design the phase modulation for RHCP and LHCP components by exploiting the P-B and propagation phase of metasurface. We adopted the Au plasmonic metasurface together with the binarized grating structure to off-axially control CVBs. Because the nano-brick needs to satisfy the half-wave condition ($\phi _x - \phi _y = \pi$), the binary grating phase is also a key point for off-axis polarization modulation using plasmonic metasurface. Due to the high reflection efficiency of the reflection-type design and the broadband response characteristics of Au and P-B phase, these CVB multiplexer/demultiplexers have high efficiency at C- and L-band. In addition, owing to the stability of Au metal, these CVB multiplexer/demultiplexers can be applied in complex environments, such as high temperature and pressure. According to the principle of optical path reversibility and mode conservation, we simultaneously multiplex and demultiplex CVBs by using the metasurfaces. It is worth mentioning that there are some aspects that could be further improved. For example, it is possible to increase the number of channels while maintaining relatively high conversion efficiency and mode purity by adjusting the structure of the vector grating (see Supplementary Note 8). It is anticipated that this off-axis polarization control method may open a new perspective for CVB applications, such as CVB multiplexing and integrated photonics. Moreover, it can be further applied to CVB holography, particle capture technology, and combine with active metasurfaces to realize dynamic off-axis control.

In summary, we proposed a metasurface-based off-axis polarization control method for CVB multiplexing/demultiplexing. We choose the CVBs with an interval of 1 to verify the feasibility of the communication system and find that the measured BERs are above the FEC threshold in the proposed communication system. Furthermore, the metasurface can be simultaneously used for wavelength-division-multiplexing, polarization-division-multiplexing, and CVB mode-division-multiplexing. We achieve a data capacity of 1.56 Tbit/s (32 × 50 Gbit/s) by multiplexing 32 channels (4 wavelengths, 4 CVB modes, and 2 polarizations) and each channel is loaded with 50 Gbit/s QPSK signals. Since the metasurface is flat and compact, it is highly promising for system integration and miniaturization. Such a technique may find applications in high-capacity communication system.

## Materials and methods

### Numerical calculations

All numerical simulations are performed by using the commercially available software FDTD Solutions (Lumerical Solutions Corp.). In the simulation, a linearly *x*-polarized plane wave (propagated along *z*-direction) with central wavelength of *λ* = 1550 nm is normally incident onto a single nanorod. In order to design the unit cell of the metasurface, periodic boundary conditions are applied along the *x*- and *y*-directions, while perfectly matching layer is imposed on the boundary along *z*-direction. The propagation phase ($\varphi _x$ and $\varphi _y$) and power reflection ($R_x$ and $R_y$) (see Supplementary Fig. S9) are obtained by sweeping the geometrical parameters of the nanopillars (width and length varying from 50 to 700 nm with an interval of 10 nm). Moreover, we could retrieve the reflection coefficients for circularly polarized light as $R_{{\rm{LL}}}\, = \,\left( {R_{xx}\, + R_{yy}\,-\,\left( {R_{yx}\,-\,R_{xy}} \right)\cdot i} \right)/2$ and $R_{{\rm{LR}}}\, = \,\left( {R_{yy}\, - R_{xx}\,-\,\left( {R_{xy}\, + R_{yx}} \right)\cdot i} \right)/2$ from the reflection of linear polarized light, where $R_{xx}$ and $R_{xy}$ represent the proportion of *x*- and *y*-polarized light, respectively. Here the number *N* of unit cell for the metasurface is set as 40 × 40 and the simulated area along the transverse plane is set as 32 × 32 μm^2^. The simulation results of metasurface at different wavelengths are shown in Fig. S10 of Supplementary Note 9.

### Fabrication of the designed metasurface

The designed metasurfaces consisting of nanoantennas are fabricated based on the standard Electron Beam Lithography (EBL). First, a thin layer of titanium (Ti, thickness: 2 nm) is deposited on the silicon substrate, which helps to increase the adhesion of the silicon (Si) substrate. Subsequently, a gold layer (thickness: 200 nm) is deposited onto the thin Ti substrate by using the electron beam evaporator (ASB-EPI-C6). Then, a SiO_2_ spacer (thickness: 150 nm) is deposited onto the gold layer substrate. After that, the positive resist film (polymethylmethacrylate (PMMA), 950 K) is spin-coated on the SiO_2_-Au-Si substrate. In order to obtain a PMMA layer with 150 nm thickness, the sample is put into the homogenizer with the speed of 4000 rpm for 1 min and then baked at 180 °C for 1.5 min. The pattern of the objective nanostructures is etched on the PMMA film by EBL (EBPG 5150) with an accelerating voltage of 100 keV and beam current of 2 nA. After etching by EBL, the sample with size of approximately $640 \times 640\, \upmu {\rm{m}}^2$ is put into the developer for 1 min and then into the fixer for 30 s. The remaining fixer is removed by nitrogen. After that, a 50 nm gold film is deposited on the sample via thermal evaporation. Finally, the sample is put in a 30% acetone solution for 6 h, where the excess PMMA and Au are removed.

## Supplementary information

SUPPLEMENTAL MATERIAL for Cylindrical vector beam multiplexer/demultiplexer using off-axis polarization control
