# Supplementary material for: Cylindrical vector beam multiplexer/demultiplexer using off-axis polarization control
Source: Light Sci Appl. 2021 Nov 2;10:222. doi: 10.1038/s41377-021-00667-7 (PMC8564545; doi:10.1038/s41377-021-00667-7)
Supplement: Supplementary file 1 — SUPPLEMENTAL MATERIAL for Cylindrical vector beam multiplexer/demultiplexer using off-axis polarization control [file 41377_2021_667_MOESM1_ESM.docx]

Supplementary Information for

**Cylindrical vector beam multiplexer/demultiplexer using off-axis polarization control**

**Shuqing Chen^1^, Zhiqiang Xie^1^, Huapeng Ye^2^, Xinrou Wang^1^, Zhenghao Guo^2^, Yanliang He^1^,** **Ying Li^1*^,** **Xiaocong Yuan^1*^, Dianyuan Fan^1^**

*Correspondence: Ying Li (queenly@szu.edu.cn) or Xiaocong Yuan (xcyuan@szu.edu.cn)*

*^1^**Institute of Microscale Optoelectronics, Shenzhen University, Shenzhen 518060, China*

*^2^Guangdong Provincial Key Laboratory of Optical Information Materials and Technology & Institute of Electronic Paper Displays, South China Academy of Advanced Optoelectronics, South China Normal University, Guangzhou 510006, China*

Supplementary Note 1: Design of binary Dammann vortex grating for generating vortex beam array

0

…

(a)

(b)


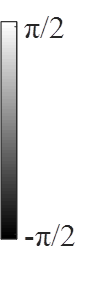


$\pi$/2

-$\pi$/2

Figure S1. (a) Phase distribution of Dammann grating in a cycle. (b) Dammann vortex grating obtained by superimposing a spiral phase on the Dammann grating

Figure S1a shows the phase distribution of a typical Dammann grating. In a cycle, its phase distribution can be expressed as [1]:

$$\Psi(x)=\sum_{k=1}^{K} \pi\times\left[rect\left(\frac{x-(x_{k+1}+x_{k})/2}{x_{k+1}-x_{k}}\right)\right]$$

where $\Psi(x_{k})$ is the phase delay, $x_{k}$ represents the phase turning point in the normalized period, $k=1,2,3,...K$, and $x_{0}=0,x_{K}=1$, *rect* is a step function. After choosing the number of diffraction orders and divergence angle of each diffraction order, we can get the optimal solution of phase turning point by using the simulated annealing algorithm. When the intensity of each diffraction order is evenly distributed, the transmission function of Dammann grating could be expressed as [2,3]:

$$\exp\left[i\Phi(x,y)\right]=\sum_{n=-\frac{N}{2}}^{n=\frac{N}{2}} \left|C_{n}\right|^{2}\exp\left[in(\frac{2\pi x}{T})\right]$$

where $T$ is the grating period, $n$ is the diffraction order from $-N/2$ to $N/2$, and $\left|C_{n}\right|^{2}=1/N$ is the power of the *n*-th order normalized with reference to the total power. As shown in Fig. S1b, to obtain the vortex beam array, we replace the term $2\pi x/T$ with $2\pi x/T+\Delta l\theta$ based on a traditional one-dimensional Dammann grating, in which $\Delta l$ is the interval of the topological charges. Hence, the transmission function of Dammam vortex grating can be expressed as:

$$\exp\left[i\Phi_{DVG}(x,y)\right]=\sum_{n=-\frac{N}{2}}^{n=\frac{N}{2}} \left|C_{n}\right|^{2}\exp\left[in(\frac{2\pi x}{T}+\Delta l\theta)\right]$$

where $\Delta l$ is the interval of the topological charges, $\theta$ is the azimuthal angle. When the incident light beam passes through the Dammam vortex grating described by Eq. S3, it is diffracted into *N* orders with equally distributed power and a topological charge of $n\times\Delta l$ in the *n*-th order direction.

Supplementary Note 2: P-B phase metasurfaces based binary vortex grating

The concept of Pancharatnam-Berry (P-B) phase was first proposed by M. Berry et al. in the United Kingdom. In 1956, S. Pancharatnam et al. found that the electromagnetic wave generates an additional phase during the polarization conversion. When an electromagnetic wave with certain polarization state evolves along a path on the surface of the Poincaré sphere and returns to the initial state, the final state differs from the initial state by a phase factor equal to half the solid angle of the closed loop [4-5]. For example, a left/right-handed circularly polarized (LHCP/RHCP) is converted to right/left-handed circularly polarized (RHCP/LHCP) beam through a half-wave plate (HWP). The resulted beam carries an additional phase, which is or , where is the P-B phase, and ,$\theta(x,y)$ is the angle between the optical axis of the HWP and the horizontal direction. Hence, when the LHCP light beam travels through the binary Dammann vortex grating, the phase response can be expressed as:

$$\Phi_{LHCP}=\sum_{n=-\frac{N}{2}}^{n=\frac{N}{2}} C_{m}\exp\left[in(\frac{2\pi x}{T}+\Delta l\theta)\right]\left[\begin{matrix} 1 \\ -i \end{matrix}\right]$$

For RHCP light beam, the phase response can be obtained by:

$$\Phi_{RHCP}=\sum_{n=-\frac{N}{2}}^{n=\frac{N}{2}} C_{n}\exp\left[-in(\frac{2\pi x}{T}+\Delta l\theta)\right]\left[\begin{matrix} 1 \\ -i \end{matrix}\right]=\sum_{n=-\frac{N}{2}}^{n=\frac{N}{2}} C_{n}\exp(-in\frac{2\pi x}{T})\exp(-i\Delta n\theta)\left[\begin{matrix} 1 \\ -i \end{matrix}\right]$$

From Eq. S5, it should be noted that both the grating phase $\exp(in2\pi x/T)$ and spiral phase $\exp(i\Delta n\theta)$ carry a negative sign compared to Eq. S4. In other words, the arrangement direction of diffraction order and the interval of the topological charges are reversed at the same time. Therefore, the topological charges of vortex beam at each diffraction order remain unchanged both for LHCP and RHCP light beam, respectively. This also explicitly indicates that CVB (de)multiplexing cannot be achieved by the P-B phase based Dammann vortex grating.

Supplementary Note 3: Independent phase control of LHCP and RHCP components by combining propagation and P-B phase


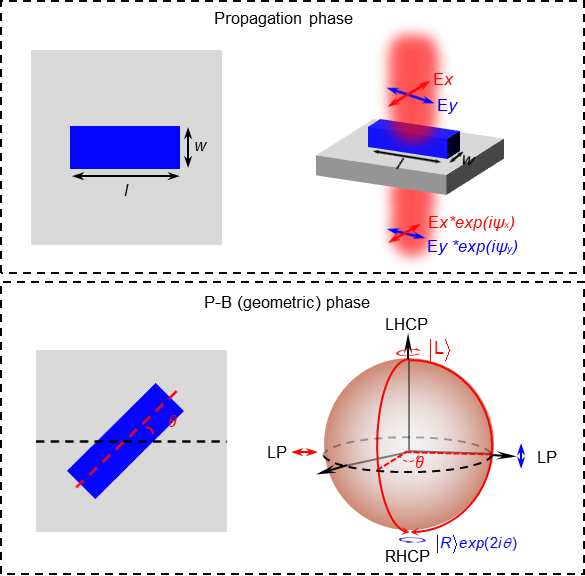


Figure S2. Principles of propagation phase and P-B phase modulation by metasurfaces

Optical metasurfaces, which are composed of subwavelength phase-shifting elements, provide an unprecedented platform for flat and integrated optics. A distinguishing feature of optical metasurfaces is the individual unit structure can be independently engineered to realize complex functionalities. Propagation phase and P-B phase are the two most common phase modulations of optical metasurface [6-7]. As shown in Fig. S2a, when a light beam passes through a metasurface composed of two media with different refractive indices, the reflection and propagation coefficients are then dramatically changed because the boundary conditions are modified by the resonant excitation of an effective current within the metasurface. The reflection and propagation waves gain a phase change varying from $-\pi$ to $\pi$. When the wavelength of the incident light and the refractive index of the nano-antenna are fixed, the phase change mainly depends on the size of the nano-antenna and the polarization state of the incident light. Here, we assume that the incident light beam polarized along *x*- and *y*- direction carries a phase change $\psi_{x}$ and $\psi_{y}$, respectively.

Variations in phase of the propagation phase metasurfaces are based on varying antennas geometry. On the other hand, the P-B phase metasurfaces achieves a full phase control by adjusting the orientation angle of antennas with identical geometry. The P-B phase is a kind of polarization-dependent phase. In other words, the phase change stems from polarization change. As shown in Fig. S2b, if two parts of a uniformly polarized wave-front are transported to a common polarization state along two different paths on the Poincaré sphere (polarization state space), a relative phase emerges between the two equal to half the solid angle ($\psi$) enclosed by the path. For example, the metasurface composed of half-wave plate elements (), it can convert the RHCP (LHCP) to LHCP (RHCP) and imposes a phase $2\psi$ to it.


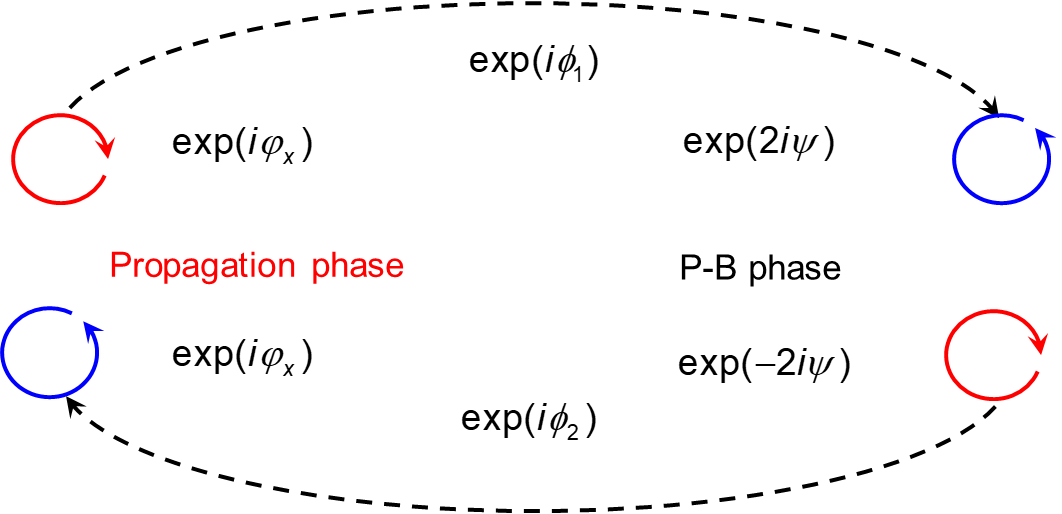


Figure S3. Principle of independent control the phase of RHCP and LHCP components by combining propagation phase and P-B phase.

In order to realize independent control of the LHCP and RHCP components of incident beam, we combine the propagation phase together with the P-B phase. The principle is schematically shown in Fig. S3, where we assume that the transmission fuction for LHCP and RHCP are and , respectively. In order to satisfy the polarization change from LHCP (RHCP) to RHCP (LHCP), the metasurfaces should satisfy half-wave condition, that is . For the propagation phase, the phase difference between and is fixed, so we can only consider one of the components. Hence, we can get the following relationship:

where is the propagation phase, and $\pm2\psi(x,y)$is the P-B phase, $\psi(x,y)$ is the orientation angle of optical axis of structural units. By solving the Eq. S6 and Eq. S7, we can get:

Hence, we can independently modulate the phase of LHCP and RHCP beam by adjusting the propagation phase  (dependent on the size of the unit structure) and P-B phase $\pm2\psi(x,y)$ (dependent on the azimuth angle of the unit structure).

Supplementary Note 4: Experiment setup for characterizing the produced CVBs


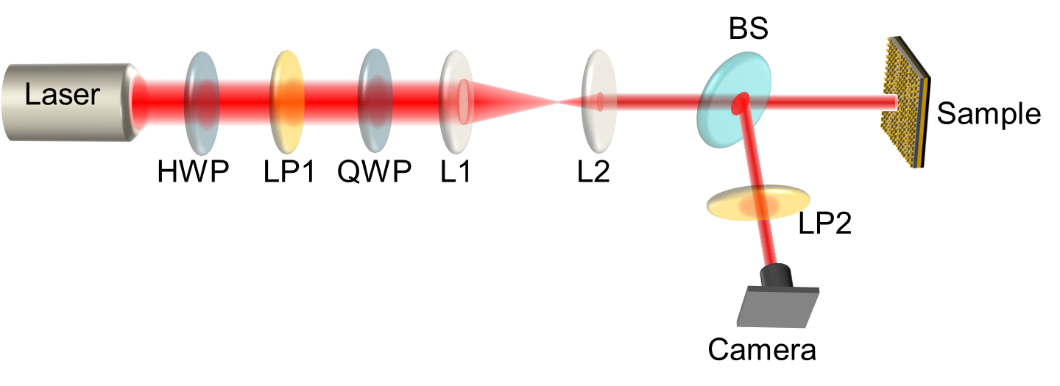


Figure S4. Experiment setup for characterizing the produced CVBs. HWP: half-wave plate; LP: linear polarizer; QWP: quarter-wave plate; L: lens; BS: beam splitter.

The experimental setup is schematically shown in Fig. S4. A laser beam with working wavelength of 1550 nm is emitted from a tunable laser source (Thorlabs, HNL210L-EC), and filtered by a linear polarizer (LP) to ensure linear polarization. The half-wave plate (HWP) between source and LP is used to control the light intensity of incident beam. Moreover, by changing the angle between quarter-wave plate (QWP) and LP, we can control the polarization state of the incident beam. The lens1 (L1) and lens2 (L2) with different focal lengths form a 4f system, which is used to determine the spot size of the incident beam. The focal lengths of lens L1 and L2 are chosen as 100 mm and 25 mm respectively, which ensures that the spot size is reduced to a quarter of the original spot size. The narrowed light beam is then incident directly on the metasurface. The reflected light passes through a beam splitter (BS) and is detected by a Charge Coupled Device (CCD) camera (Ophir, SP928).

Supplementary Note 5: Optical characterization of metasurface in 1310 nm band


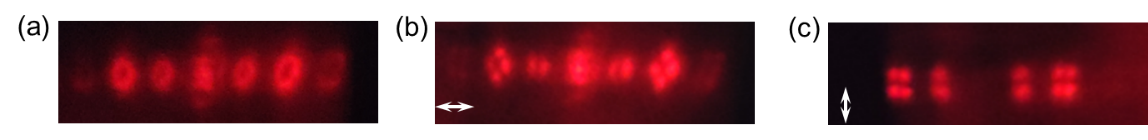


Figure S5. (a) Far-field distributions of the light beam with 1310 nm wavelength through metasurface. (b) and (c) Images of metasurface-generated CVBs with horizontal and vertical linear polarizer, respectively.

In order to further estimate the effective bandwidth of the metasurface based CVB (de)multiplexer, here we use a laser with working wavelength of 1310 nm as the light source. As shown in Fig. S5, when a Gaussian beam with wavelength of 1310 nm is incident and reflected by the metasurface, the far-filed light intensity distribution is shown in Fig. S5a. Due to the limitation of experimental conditions, our CCD camera does not support beam imaging at 1310 nm. Hence, here we use a handheld photosensitive card (Thorlabs, VRC2) to detect the light intensity distribution, and use a phone to record the picture. Figures S5b and S5c show the measured intensity profiles with a linear polarizer placed in front of the handheld photosensitive card. The white double-headed arrows show the direction of the polarizer’s transmission axis. From the measured intensity profiles in Figs. S5a - S5c, it can be proved that the metasurface can produce multiple off-axis CVBs well at 1310 nm. These results further verify that this metasurface has a broadband response in CVB multiplexing and demultiplexing.

Supplementary Note 6: Schematic diagram of communication experiment


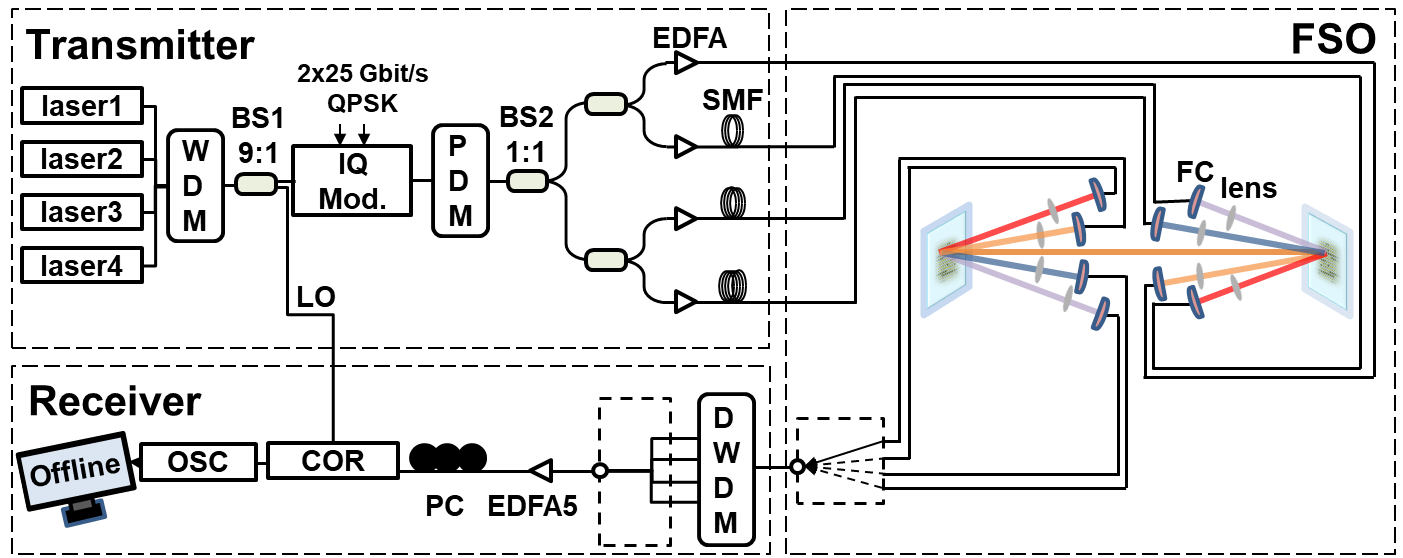


Figure S6. Schematic diagram of the CVB multiplexing communication system. I-Q Mod.: I-Q modulator; PPG: programme pulse generator; OC: optical coupler; EDFA: Erbium-doped fiber amplifier; SMF: single mode fiber; FC: fiber coupler; PC: polarization controller; COR: coherent optical receiver; OSC: oscilloscope.

Figure S6 shows a schematic diagram of the experimental set-up for CVB multiplexing and demultiplexing. At the transmitting end, the Gaussian beam emitted by the laser is divided into two sub-beams by the optical coupler (OC1 (9:1), 90% are carrier beams and 10% are local beams). The quadrature phase-shift keying (QPSK) signal with 50 Gbit/s capacities is generated by the programme pulse generator (PPG) and then loaded onto the signal beam through the IQ modulator. The generated optical signals were coded to two polarizations by utilizing a polarization-dependent beam splitter (PBS), an optical delay line and a polarization beam combiner (PBC). The signal light beam is divided into four sub-beams by using OC2 (1:1), OC3 (1:1), and OC4 (1:1). Then the sub-beams are amplified by an erbium-doped fiber amplifier (EDFA), and each of the sub-beams is delayed by a single mode fiber (SMF) with relatively large length for data-pattern decorrelation. These signal light beams are coupled from the fibers to free space through fiber collimators. Before the light beams pass through the metasurface at different angles, we use a 4*f* system composed of two lenses to reshape the light beam to ensure that it is smaller than the metasurfaces. Then the incident Gaussian fundamental modes carrying different signals are transformed into CVBs with different polarization orders (related to incident angle) and coaxially transmitted along with the zeroth diffraction order. After propagation in free space over a distance of 1 m, the beams with the multiplexed CVB channels are demultiplexed by using another metasurface with exactly the same parameters. When passing through the demultiplexing grating, the coaxial CVBs are diffracted into 4 orders. The signal beam with a polarization order induced at a certain diffraction angle is compensated and converted into a Gaussian beam with a bright high-intensity spot at its center. Beams with other polarization orders are ring-shaped, which have low intensity in the center. We use additional pinhole apertures to filter out these rings. The focused Gaussian beam is then coupled into a fiber by a microscopic objective and respectively amplified by an erbium-doped fiber amplifier. After adjusting the polarization state through the polarization controller, the signal and local beams enter the coherent receiver together for coherent demodulation, which is sampled by a sampling oscilloscope. The sampled data is processed offline by a computer.

Supplementary Note 7: Compatibilty of the metasurface with wavelength-division-multiplexing


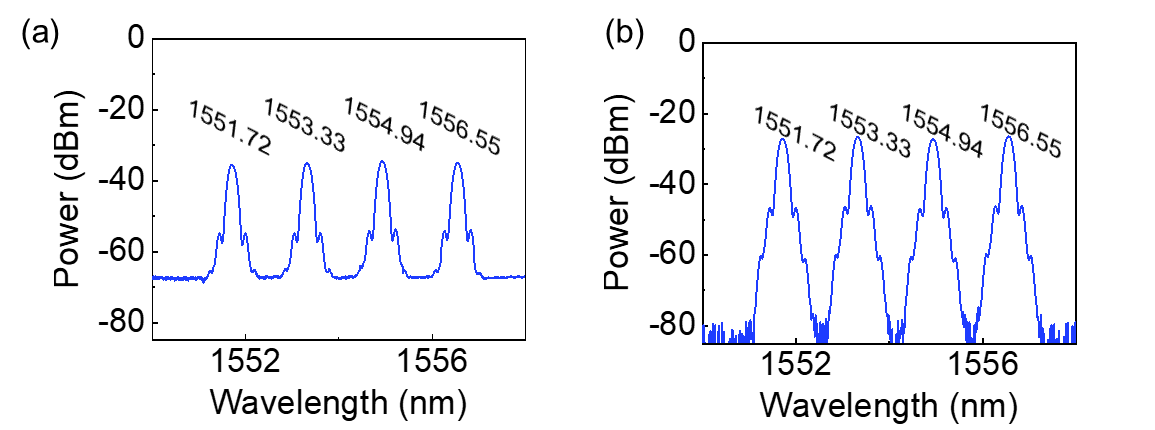


Figure S7. Optical spectrum (a) before free-space transmission and (b) after free-space transmission.

In order to ensure that the CVB (de)multiplexer is compatible with wavelength-division-multiplexing, the most important feature is that the metasurface will not broaden the linewidth of incident wavelength and generate crosstalk between wavelengths. To verify the compatibility of our metasurface with wavelength-division-multiplexing, we measured the optical spectrum of wavelengths 1551.72, 1553.33, 1554.94 and 1556.55 nm before and after multiplexing and demultiplexing as shown in Figs. S7a and S7b, respectively. From the optical spectrum, the linewidth of resonant peaks is broadened due to the power of signal is amplified by Erbium doped fiber amplifier (EDFA). However, the peak wavelength is not shifted after propagating through the metasurface in free space optical communication, which indicates that in the wavelength-division-multiplexing system, the metasurface has low dispersion and broadband. These results explicitly confirms that the metasurface based CVB (de)multiplexer is compatible with wavelength-division-multiplexing.

Supplementary Note 8: Method to increase the number of multiplexed channels


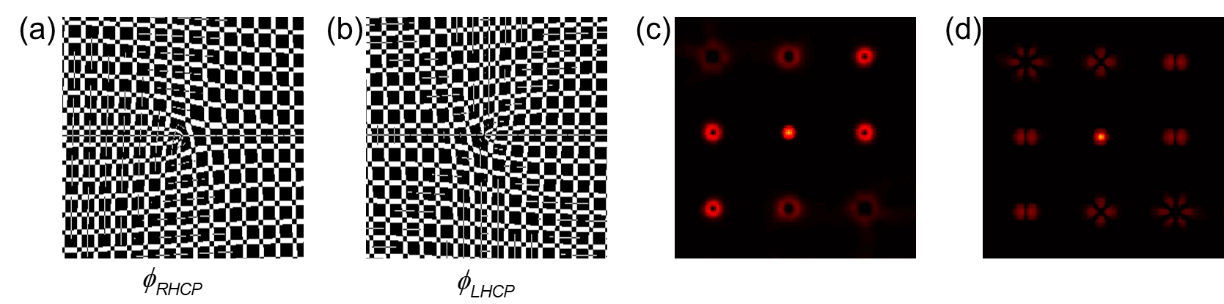


Figure S8. Further increase the number of channels by using the two-dimensional (2D) Dammann gratings. (a) Phase response of RHCP incident beam. (b) Phase response of LHCP incident beam. (c) Optical intensity distribution of the Gaussain beam reflect by 2D vector Dammann grating. (d) Optical intensity distribution after passing through the polarizer.

In order to further increase the channel capacity, one of the potential methods is to expand the number of multiplexed CVBs. Here we initially verify the feasibility of this method by numerical simulation. The transmission function of two-dimensional Dammann vortex gratings can be expressed as:

$$\exp\left[i\Phi_{2D-DVG}(x,y)\right]=\sum_{a=-\infty}^{a=+\infty} \sum_{b=-\infty}^{b=+\infty} \left|C_{ab}\right|^{2}\exp\left[ia(\frac{2\pi x}{T_{x}}+\Delta l_{x}\theta)+ib(\frac{2\pi x}{T_{y}}+\Delta l_{y}\theta)\right]$$

where $\left|C_{ab}\right|^{2}$ is the energy of each diffraction order normalized to the total power, $\Phi_{2D-DVG}(x,y)$is the phase distribution of two-dimensional Dammann vortex gratings, $T_{x}$ and $T_{y}$ are the grating periods in the *x*- and *y*-directions, respectively. $\Delta l_{x}$ and $\Delta l_{y}$ are the interval of the topological charges in *x*- and *y*-directions. The phase response of LHCP/RHCP with two-dimensional Dammann vortex gratings (the topological charges at same diffraction order in the far-field distribution are opposite) has been shown in Figs. S8a and S8b, respectively. In this instance, when a linearly polarized Gaussain beam illuminates the metasurface, the far-field light distribution is shown in Fig. S8c. After passing through the polarizer, as shown in Fig. S8d, the doughnut shape intensities are split into multiple light spots and the number of light spots is twice than the polarization order of CVB. It confirms that the generated beams are CVBs with non-uniform polarization distributions.

Supplementary Note 9:


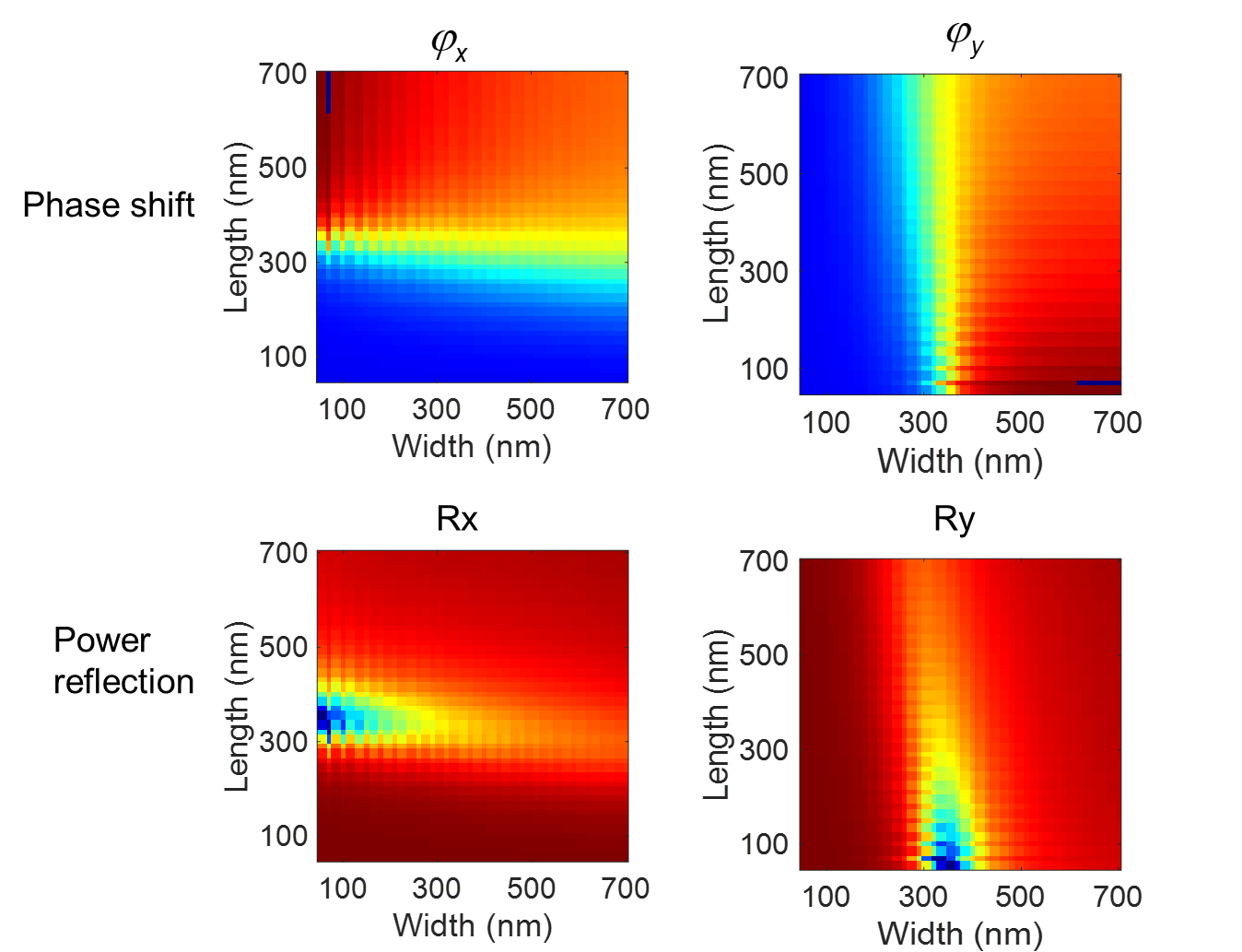


Figure S9. Simulation results for two-dimensional parameter sweeps of Au pillars (*h* = 50 nm).

The phase shift () and power reflection ($R$) of *x*- and *y*-polarization light scattered from were numerically characterized using finite difference time domain (FDTD) simulations as a function of the nanoantennas length, width at a wavelength of 1550 nm has been shown in Fig. S9. Due to the phase of the grating we used is binarized, with only “0” and “$\pi$”. Under this condition, through the calculation of Eq. S9, the propagation phase only three states of “0”, “$\pi/2$”, “$\pi$”. In order to ensure that spin transformation from LHCP (RHCP) to RHCP (LHCP), here we choose to first find the point of satisfying . In this case, we further search for satisfaction among these points , respectively. The appropriate nanoantennas dimensions are S1 (100 nm, 340 nm), S2 (220 nm, 380 nm), S3 (260 nm, 580 nm), And the polarization conversion efficiency of S1, S2, S3 are 0.93, 0.73, 0.91, respectively. Finally, by combining the length (*l*) and width (*w*) determined by and the rotation angle determined by $\psi(x,y)$, we can obtain the structure distribution of the entire metasurface based CVB (de)multiplexer.

In order to verify the feasibility of our scheme, we simulate the designed whole metasurface by using the method of FDTD. Due to the limitation of simulation conditions, here we set the number N of unit cell in the cross-section as 40×40, the transversely simulation area is set as 32×32$\mu m^{2}$. The top row of Fig. S10 shows the light intensity distribution obtained in the far field after linearly polarized Gaussion beam normal reflected by the metasurface at different working wavelength (1550, 1600, 1650 nm). The bottom row of Fig. S10 shows the doughnut shape intensities are split into multiple light spots and the number of light spots is twice than the polarization order of CVB. It confirms that the generated beams are CVBs with non-uniform polarization distributions.


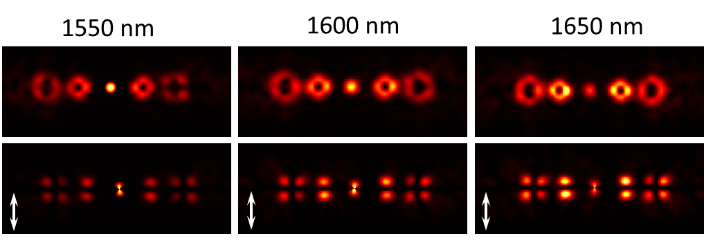


Figure S10. Simulation result of the metasurface at different wavelengths (1550, 1600, 1650 nm). Top row: the optical intensity distributions obtained in the far field after linearly polarized Gaussion beam normal reflected by the metasurface. Bottom row: the optical intensity distributions of metasurface-generated CVBs pass though vertical linear polarizer.

**References**

[1]C. Zhou, and L. Liu, “Numerical study of Dammann array illuminators”, *Appl. Opt.*, **34**(26), 5961-5969 (1995).

[2] L. Janicijevic, S. Topuzoski, “Fresnel and Fraunhofer diffraction of a Gaussian laser beam by fork-shaped gratings”, *J. Opt. Soc. Am. A*, **25**(11), 2659-2669 (2008).

[3] Y. Fu, C. Min, J. Yu, Z. Xie, G. Si, X. Wang, Y. Zhang, T. Lei, J. Lin, D. Wang, H. P. Urbach, X. Yuan, “Measuring Phase and Polarization Singularities of Light Using Spin-Multiplexing Metasurfaces”, *Nanoscale*, **11**, 18303–18310 (2019).

[4] L. Marrucci, C. Manzo, D. Paparo, “Optical Spin-to-Orbital Angular Momentum Conversion in Inhomogeneous Anisotropic Media”, *Phys. Rev. Lett.*, **96**(163905), 1-4 (2006).

[5] G. Milione, H. I. Sztul, D. A. Nolan, R. R. Alfano, “Higher-Order Poincare´ Sphere, Stokes Parameters, and the Angular Momentum of Light”, *Phys. Rev. Lett.*, **107**(053601), 1-4 (2011).

[6] M. Berry, “Quantal phase factors accompanying adiabatic changes”, *Proc. R. Soc. A*, **392**, 45 (1984).

[7] S. Pancharatnam, “Generalized theory of interference and its applications”, *Indian Acad. Sci. A*, **44**(247), 398-417 (1956).
